# Experience of compassionate care in mental health and community-based services for children and young people: facilitators of, and barriers to compassionate care– a systematic review

**DOI:** 10.1007/s00787-025-02711-y

**Published:** 2025-04-04

**Authors:** Frane Vusio, Kathryn Odentz, Charlene Plunkett

**Affiliations:** School of Health in Social Sciences, Old Medical School, The University of Edinburgh, Elsie Inglis Quadrangle, Teviot Pl, Edinburgh, EH8 9AG UK

**Keywords:** Children and young people, Compassionate care, Experience and satisfaction, Parental experiences, Staff experiences, Mental health and welfare

## Abstract

Compassion and compassionate care for children and young people (CYP) encompass a variety of emotions, including sympathy, empathy, and sadness for their suffering, alongside actions intended to alleviate their distress. While compassion is a well-recognised concept in health and social care, literature has identified various barriers and facilitators that affect the delivery of compassionate care. These include compassion fatigue, time limitations, organisational and clinical culture, insufficient resources or support, technological advancements, and burdensome administrative tasks. Despite being viewed as essential in health care, the concept of compassionate care remains poorly defined and expressed, particularly in the context of mental health services for CYP. This review explored the perspectives of CYPs, their parents, and staff regarding the compassionate care provided in community services. Additionally, it aimed to identify factors that facilitate or obstruct compassionate care for CYPs in both mental health and community settings services. From June to August 2024, a search was carried out for peer-reviewed articles and grey literature, with additional searches conducted in October 2024. The initial search produced 7,461 articles, with 23 selected for this review. A thematic synthesis organised the studies based on the main themes identified: ‘Compassionate care is all about humanity’ and ‘the complex interplay of facilitators and barriers to compassionate care’. The identified articles highlighted the increasing need for compassionate care in community-based services for CYP, along with the range of facilitators and barriers to providing this care. Lastly, we propose an alternative definition of compassionate care tailored to these services.

## Introduction

Compassion is now widely recognised as a crucial aspect of healthcare provision worldwide [1, 2]. Similarly, compassionate care is fundamental to the UK’s NHS constitution and a crucial component of the UK’s healthcare system [3]. Compassion is defined mainly as the strong sensation of empathy, sympathy, and sadness for individual suffering, accompanied by a desire to relieve that distress or suffering [1, 4].

However, determining a precise definition of compassion is challenging because it shares similarities with other constructs, such as empathy or sympathy [5], with the former defined as the capacity to resonate with another’s emotional state [6–8]. Empathy and compassion differ in that empathy involves feeling others’ suffering with them, while compassion involves feeling for their suffering [5]. In contrast, sympathy can be defined as an emotional response of concern toward the suffering of others [9].

Nevertheless, empathy and sympathy are all considered part of the compassion-related states [10]. Compassion differs from these concepts in several aspects, particularly in its association with suffering, vulnerability arising from engaging with suffering, psychological motivation, reciprocal and experiential nature, and focus on action [7].

Action is a crucial element of compassion, often absent in empathy or sympathy [10]. As such, taking action is essential for demonstrating compassion and providing care [1]. A review defined compassionate care from patients’ point of view as “*a virtuous response addressing suffering through relational understanding and action*,” while healthcare staff saw it as “*an intentional response to know a person*,* discern needs*,* and ameliorate suffering through relational understanding and action*” [7]. Even with varying perspectives on compassion, compassion is vital in healthcare delivery, involving recognising issues, problem-solving, and empathy—all essential for recovery [1].

However, compassion can also be considered a controversial, multifaceted, and sensitive concept due to the complex interplay of evolutionary, philosophical and sociological theories [10, 11]. For instance, evolutionary theory defines compassion as facilitating cooperation [12] and care for vulnerable individuals [13]. Interestingly, the philosophical definition of compassion is more in line with evolutionary theory. For example, compassion is associated with a concern for others and their suffering [5]. All humans experience deep suffering; therefore, compassion involves actively engaging with others to alleviate or reduce suffering rather than perceiving it merely as a state of empathy [5]. Both theories allude to crucial elements of compassionate care, namely action and care. Therefore, compassionate care involves recognising and addressing the suffering and unmet needs of others [14, 15].

Conversely, some authors define compassion through sociological lenses as “*intelligence*” in providing evidence-based care while advocating for justice, contributing to an empathetic response aligned with the culture and the local community [16]. Indeed, compassion is fostered by social identity, with cultural differences influencing its expression [13]. Culture, encompassing shared norms, values, and beliefs within a specific geographical area, greatly influences the development of compassion, while a society’s interpretation of distress or suffering shapes compassionate action and care [5].

A previous review highlighted cultural differences influence how compassion is expressed and experienced [17]. For example, in East Asia, collectivistic societies may instil cultural expectations and social pressures that emphasise group harmony over individual emotional openness [18]. In turn, help-seeking behaviours may be viewed as a weakness that brings shame to the individual and their family or community, potentially creating barriers to compassion being sought or offered [18]. Furthermore, a study comparing perceptions of compassion in Singaporeans and Australians identified notable differences in how compassion and fears of compassion interrelate [19]. However, while cultural differences shaped the way compassion was expressed and experienced, its overall impact on psychological well-being appeared to be consistent across these two groups [19]. These findings highlight the intricacies of defining compassion due to the role of cultural and societal factors on its expression and internalisation. When implementing compassion-focused interventions and Western practices in non-Western settings, it is essential to modify them to align with the cultural norms and values of the intended audience [18].

Indeed, compassionate care does not exist in isolation. The system in which professionals deliver care is crucial in delivering compassionate care [5]. Therefore, compassionate care has been viewed as more than just an appropriate action or response. Instead, it has been perceived as “*a virtuous and emotionally intelligent response to suffering*” [20].

Nevertheless, despite differing perspectives on what constitutes compassion and compassionate care, which may stem from cultural differences in the perception of compassionate behaviours, the fundamental element of compassion care lies in its response (action) and willingness to aid those experiencing distress. Compassionate care is especially vital when supporting those facing mental health challenges, as an absence of compassion can deeply affect their well-being and recovery [5].

Compassionate care is essential as it enhances patient experience, satisfaction, and clinical outcomes [21]. It also positively impacts the quality of life [22–24], reduces disease burden [7], enhances the quality of care [25], and improves the sustainability of healthcare systems [26]. Numerous studies have recognised the absence of compassionate care in healthcare services, highlighting a pressing need for it [27]. A lack of compassionate care is associated with decreased satisfaction among patients and staff [20, 28, 29], reduced treatment adherence [21], negative treatment outcomes [30, 31], and increased healthcare costs [26].

Furthermore, some systematic reviews indicate that education can improve clinicians’ ability to provide compassionate care by allowing them to learn from role models [7, 32]. Various studies have pinpointed a combination of individual, team, and organisational factors as essential enablers of compassionate care [1, 33]. Additionally, the emotional ties clinicians have with the health system, their feeling of being appreciated and supported, and the availability of practical assistance further enhance compassionate care [1].

Nonetheless, healthcare is widely acknowledged as an extremely stressful environment [34]. Healthcare and welfare workers’ stress is a major obstacle to providing compassionate care and a key factor in compassion fatigue [1]. Additionally, work-related stress and physical and emotional fatigue can result in a state of exhaustion, marked by a lack of compassionate care and a diminished ability of healthcare professionals to express empathy, sympathy, or compassion towards their patients [1, 35]. A recent systematic review identified additional barriers, such as time constraints, organisational and clinical cultures, insufficient resources or practical support, advancements in technology, and burdensome administrative demands, which may result in compassionate care being perceived merely as a checklist activity [33].

Indeed, various evidence indicates the significant impact of compassion fatigue on systemic, organisational, and individual factors that may hinder compassionate care. Nevertheless, individual, team, and organisational factors can likely serve as both barriers and facilitators of compassionate care [1].

Evidence for compassionate care in the mental health system remains an underexplored area [27, 33]. However, key stakeholders, such as policymakers, clinicians, researchers, educators, and service users, show significant interest in compassion in healthcare, especially since compassion is crucial in providing good-quality mental healthcare [33]. Although compassion is recognised as a vital aspect of health care, its application in CYP mental health services is still poorly defined or understood [1]. Besides, a gap exists in understanding the barriers and facilitators to compassionate care [1].

Moreover, numerous studies have underscored the presence of a compassion crisis in healthcare, especially within mental health services [5]. Most studies on compassionate care mainly focus on adults and staff [20, 27, 33]. A literature review identified just one study focusing on staff experiences of compassionate care in adolescent mental health wards [1]. Currently, no defined studies or systematic reviews explain compassionate care for CYP, as well as their families and caregivers. Hence, a systematic review was performed to consolidate existing evidence and gain insights into compassionate care within CYP services to fill this gap.

Therefore, this systematic review aims to answer the following research question: “*What is the current state of knowledge on the delivery of compassionate care in children and young people’s mental health and community-based services*?” The review also aims to understand CYPs’, their carers’, and staff’s perceptions of compassionate care delivered in community-based services and what factors facilitate or hinder compassionate care for CYPs in mental health and community-based services.

## Methods

This systematic review was conducted and reported following the PRISMA guidelines [36]. The protocol for this review was submitted and approved by PROSPERO (ID: **CRD42024548623**).

### Search strategy

**Fig. 1:**
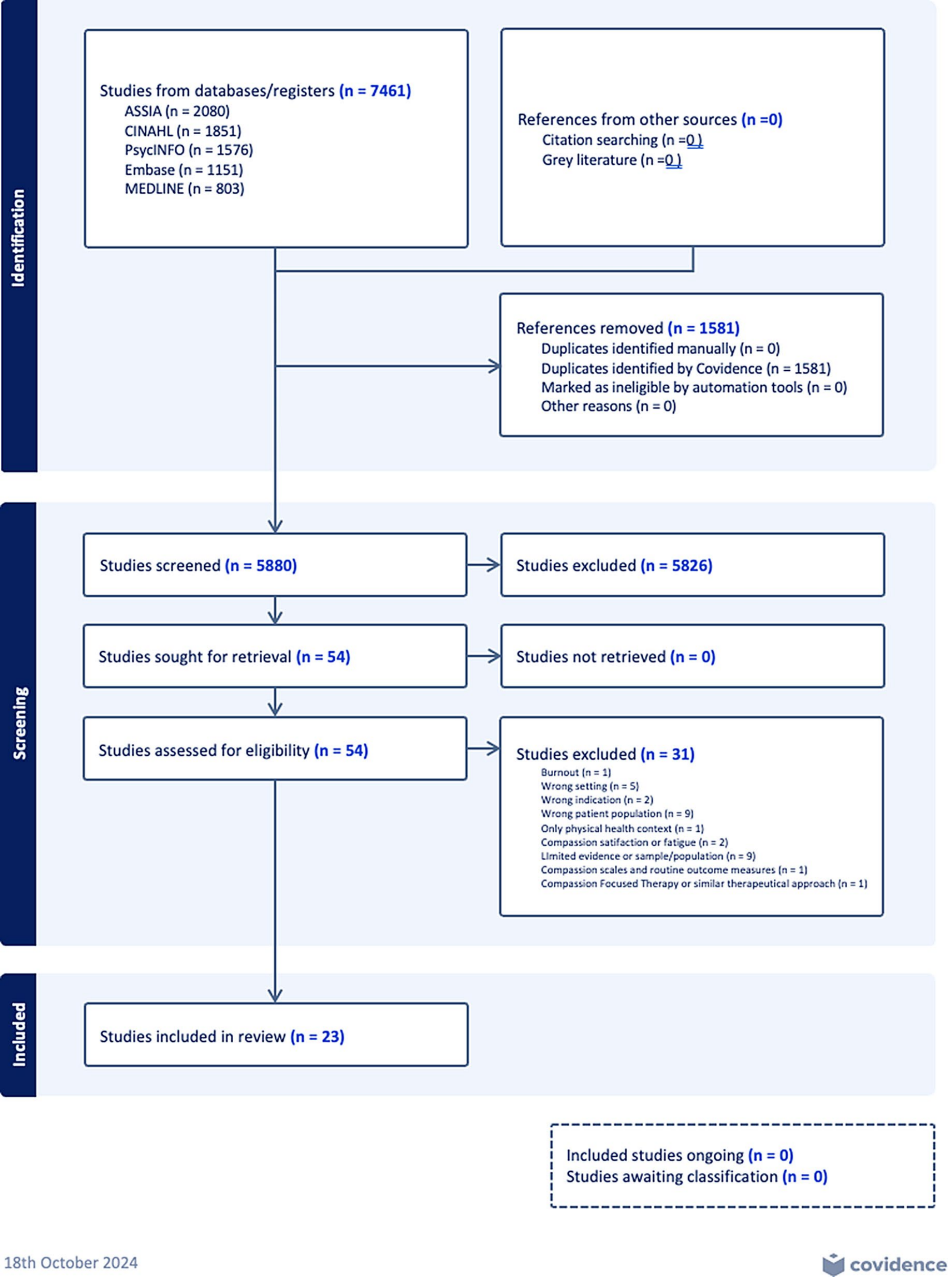
PRISMA flowchart

The search strategy was shaped and modified based on previous scoping and systematic reviews [7, 20, 32, 33]. This process involved collaboration with the Social Science librarian utilising the Population, Interest, and Context (PICo) framework, along with the relevant key terms and synonyms: ‘*children and young people’*, ‘*compassion*’, ‘*patient-centred’*, and ‘*community-based settings’*. The search strategy illustrated in Fig. 1 was executed across the PsycINFO, Embase, Medline, ASSIA, and CINAHL databases. The latest search was conducted in October 2024; however, it did not produce any new articles. Furthermore, we carried out both forward and backward manual searching on the articles that satisfied our inclusion criteria. The forward-searching method allowed us to discover recent works by authors of these qualifying studies, while the backward strategy involved reviewing the references cited within those articles.

**Table 1:** PICo framework and searching strategy

PICo framework	
Population (P)	(Child* OR adolescen* OR young pe* OR emerging adult* OR youth* OR juvenile* OR minor* OR teen* OR young adult*).tw
Phenomena of interest (I)	(compassion* OR sympathy* OR empath* OR client cent* OR client focus* OR patient focus* OR patient cent* or person focus* OR person cent* OR caring approach).tw
Context (C)	(((mental health* OR psychology* or psychotherapy* OR communit*) adj3 (clinic* or service* or care or facility or facilities)) OR CAMHS or “residential care” or foster* or “respite care”).tw

### Eligibility criteria

As the term ‘young person’ can differ by geographical and healthcare settings, we initially considered the World Health Organization’s definition, which categorises young people as those aged 10–24 [37]. However, as some CYP mental health service models cater to individuals aged 0–25, we extended the upper age limit to 25. This modification enabled us to include literature relating to compassionate care experiences from these models, while also encompassing alternatives (e.g. 14–25, 0–19) and traditional mental health service models that cater to 0–18 year olds.

Assessing the articles’ suitability with inclusion and exclusion criteria ensured a focused search.

The inclusion criteria included individuals aged 0–25 receiving compassionate care, parents of CYP within this age range, and community-based staff from statutory, voluntary, or private mental health services. This encompasses those working in community-based urgent care, inpatient care, and residential or foster care. The review focused on qualitative and mixed-method studies.

Exclusion criteria included studies involving participants older than 25, those centred on burnout or compassion-related strategies, materials not in English (excluding Croatian, Italian, French, or Spanish), and any prior scoping or systematic reviews or book chapters.

### Study selection

Five electronic databases provided articles for analysis in Covidence. After eliminating duplicates, researchers FV and KO reviewed the titles and abstracts. FV and KO further evaluated studies that met the inclusion criteria in full text, with a third researcher, CP, available for consultation in case of any disagreements; however, none arose.

### Quality assessment and risk of bias

The Critical Appraisal Skills Programme (CASP) evaluates the quality of selected articles. This popular ten-item tool examines the quality and validity of different research studies, including qualitative ones [38]. CASP aids researchers and healthcare professionals in assessing the validity and relevance of study findings, promoting evidence-based practice and critical thinking [39]. Researchers FV and KO assessed each study based on CASP criteria. Any disagreements were resolved through further discussion.

### Data extraction

The data extraction form was created and tested on a limited research sample before its implementation across all relevant studies. Our results are categorised into four main groups, with key data extracted including: authors, publication year, country of origin, study aims, study design, population, interests, and context. Two reviewers (FV and KO) independently retrieved the data.

### Data synthesis

The thematic synthesis protocol established by Thomas and Harden (2008) was used in this review [40]. The purpose of thematic synthesis is to enhance understanding by creating broad themes beyond just the original studies’ descriptive aspects [41].

Thematic synthesis follows a three-step process. First, researchers conduct line-by-line coding of findings from primary studies. Next, they generate ‘descriptive themes’ and ‘analytical themes’ in the final step, resulting in new interpretive accounts [40]. In the third stage, researchers explore beyond primary descriptive themes that align with the original findings [40]. FV and KO independently analysed descriptive themes and identified barriers and facilitators to compassionate care. Initially, each researcher interpreted the themes separately before aligning their interpretations through discussion. While developing more abstract themes, they carefully considered the context of the original studies [42].

Noyes and Lewin (2011) state that the iterative method of analysing qualitative data involves multiple readings of primary studies, data extraction, and qualitative synthesis, leading to the identification of new themes [43].

## Results

**Fig. 2:**
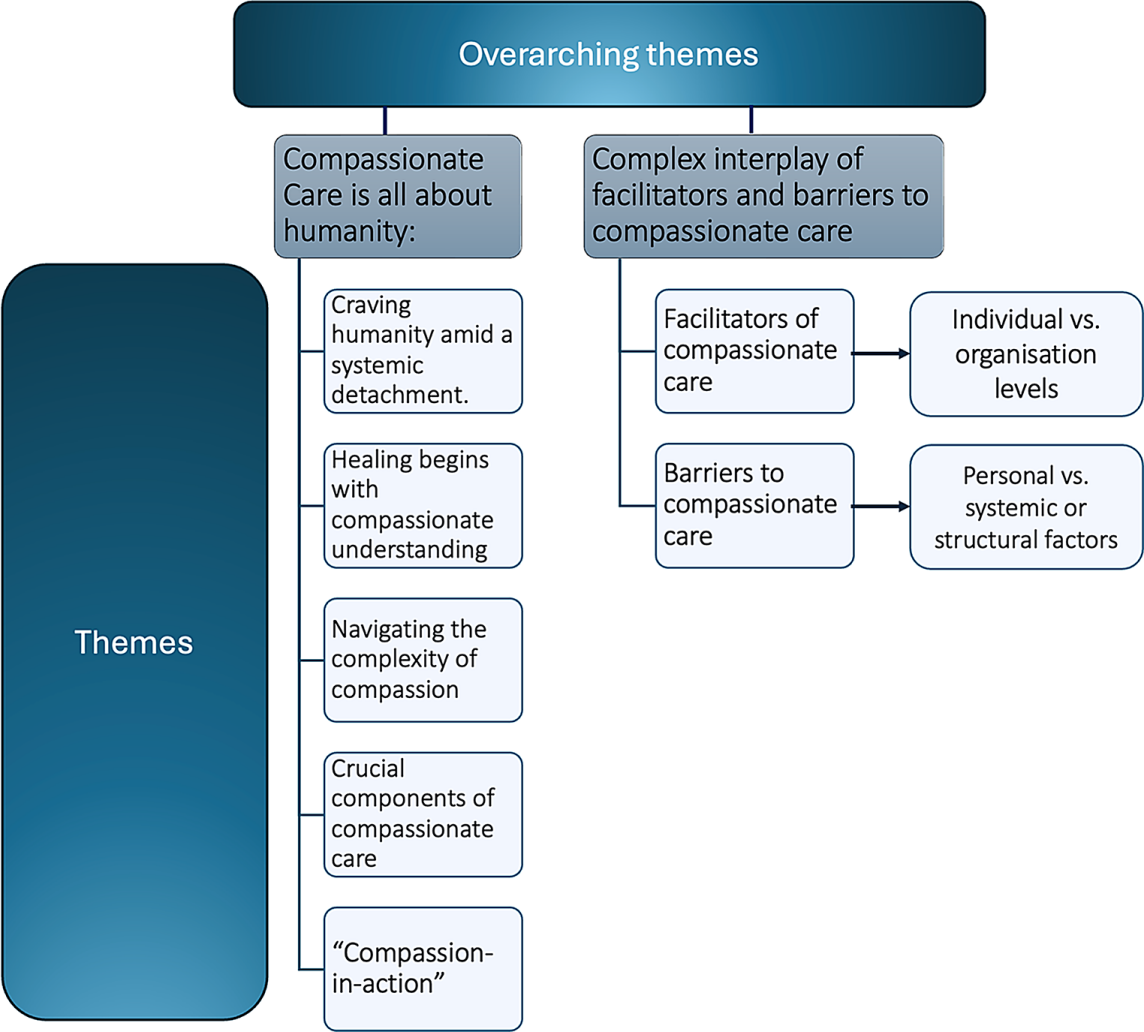
Thematic synthesis map

The search strategy identified 7461 articles. After removing duplicates, 54 papers were selected for examination, while 5818 studies were excluded. Common reasons for exclusion included focus on physical health, adult populations, compassion fatigue or burnout, and lack of relevance to CYP mental health. Of the 54 screened papers, 23 were selected for in-depth review by both raters, with no disagreements. The full process is shown in the PRISMA flowchart (Fig. 2).

### Study characteristics

The 23 studies included in this review came from nine different countries: seven from the USA [44–50], nine from the UK [51–59], one from Portugal [60], two from Canada [48, 61], one from China [62], Australia [63], Ireland [64] and Germany [65].

Seventeen studies used qualitative methods [44, 46, 47, 49–52, 55–58, 60, 62–65]; two were based on a qualitative case-study approach [44, 48], while two had a mixed-method design [54, 66]. The remaining two studies used other designs, such as conceptual reviews and cross-sectional designs [45, 61].

The articles included were published between 2009 and 2024, with most published in the last four years. Ten studies conveyed experiential data from CYP [45, 50, 54, 55, 58, 61, 63, 65], eight from parents and carers [44, 48, 52, 56, 59, 62, 66], while the remaining 16 studies encapsulated the experiences of staff or professionals’ compassionate care when working with CYP/parents [44, 46–49, 51–53, 56–58, 60, 62, 64]. The sample sizes of participants in the included articles ranged from 4 to 18,717. Table 1 summarises the 23 included studies and their characteristics.

### Quality appraisal

We categorised the studies into three quality groups: low, medium, and high [38]. A study was high quality if it met 80%-100% of the ten criteria, marked as ‘Yes’ (Y). Those meeting 50%-80% were rated medium quality, indicating partial fulfilment of criteria. Studies meeting 50% or fewer were labelled low quality, implying they met only minimum requirements. Unclear studies were marked ‘Unclear’ (?) and did not meet the criteria (N). The review included all studies regardless of quality, per Marshman et al. (2024) recommendations. Three studies were classified as low-quality [45, 48, 61], and three had moderate quality [44, 47, 64]. The remaining seventeen studies were of high quality. Details of the quality appraisal can be viewed in Appendix 1.

### Synthesis of results

The final sample consisted of 23 studies focused on two main themes (Fig. 2). The first theme, ‘Compassionate care is all about humanity’, reflects stakeholders’ experiences of compassionate care. The second theme examines factors that foster compassionate care, including active listening, supportive environments, and ethical resilience to promote healing. It also identifies obstacles to delivering compassionate care, such as ineffective communication and excessive workloads.

### Compassionate care is all about humanity

The first overarching theme identified was ripples of compassionate care, which comprised five themes: (1) craving humanity amid a systemic detachment; (2) healing begins with compassionate understanding; (3) navigating the complexity of compassion; (4) crucial components of compassionate care; and (5) “Compassion-in-action”.

Compassionate care is crucial in mental health and social services for the humanity of all involved: YP, parents, healthcare providers, and social workers. The narrative highlights how compassion fosters trust and connection, as well as the consequences of its absence.

### Craving humanity amid a systemic detachment

Many YP seeking or receiving support from mental health services feel that the care they receive lacks compassion, empathy, and personal attention, which makes it difficult for them to recover. A study by Loos et al. (2018) highlighted that many YP want to be seen as individuals or human beings rather than just cases. One young person in the study stated: “*I also have a life as a human being and not just as a sick person*”. When care is perceived as indifferent, paternalistic, bureaucratic, dehumanised, and detached, it can discourage YP from seeking help in the future and lead to disengagement [61, 65]. The experience of another participant from the study by Loos et al. (2018) captured this sentiment: “*One just realises that the ward round is led by people who are not really interested in their work. I do not have the impression that anybody is seriously interested. They are all so apathetic*…”. Feeling dismissed as just another task in a staff member’s routine undermines the trust and connection crucial for compassionate care [65].

### Healing begins with compassionate understanding

Parents often find themselves dealing with their emotional struggles while advocating for their children’s mental health and well-being. They stress the importance of being treated with compassion. Research conducted by two studies [48, 59] illustrates that families who experience respectful treatment feel supported and are more empowered in caring for their children. One parent shared how their experience changed when met with genuine compassion: “*She admitted she didn’t know anything about OCD and spent a lot of time on the phone letting me explain. Felt supported and that she was genuinely interested*” [59]. Another parent shared their positive experience of the practitioner who demonstrated their compassionate care “*She’s very nice. Anything she does*,* she asks you first for your opinion. The children also like her a lot. She usually first talks to the children*,* play with them a little first.*” [66]. These instances of empathetic involvement not only support their child’s mental health but also enhance the well-being of the parents [59].

Conversely, facing dismissive attitudes, arrogance, and a lack of empathy from healthcare professionals makes parents feel overlooked and unsupported. One parent recounted her experience of dehumanising care, which made her feel isolated and alone unsupported: “*The doctors*,* and sometimes the nurses*,* aren’t very nice. Sometimes*,* the doctors are arrogant and prideful. Very arrogant. Sometimes*,* when you ask the nurse something*,* they are arrogant and say you have to wait*” [66].

The theme of compassionate care is also evident through the voices of parents and families involved in child welfare. Their experiences highlight the vital role of humanity, relationships, and emotional healing in tackling the challenges confronted by families in crisis.

As suggested by Markey and Sankaran (2020), families involved in the child welfare system do not need to be ‘*fixed*’; instead, they need to be “*healed*”. This viewpoint changes the focus from simply solving problems to fostering support, recognising that families frequently find themselves in situations beyond their control. By highlighting emotional stability and comprehensive support, it dismisses the ideas of blame or shame, which can further burden individuals already struggling with the crisis [48]. By recognising the inherent dignity of parents, practitioners can foster environments that reduce stress and promote healing [48].

Additionally, it is essential to acknowledge the significance of compassion and positive interaction for supporting family well-being. As highlighted by another parent in Markey and Sankaran’s study (2020),” *It is also essential to fulfil their [parents] basic human need to experience compassion*,* understanding*,* and positive engagement. Recognising and tending to families’ emotional needs*,* in my opinion*,* is more effective than providing concrete goods or funds*”. Families experiencing crisis confront both practical hurdles and emotional distress; empathetic support provides essential stability in such moments. These insights underscore the significance of compassion and humanity in caregiving and welfare. Recovery and healing extend beyond merely delivering services or addressing immediate concerns; they encompass fostering relationships that honour the emotional well-being of patients, service users, and families, empowering them through trust, dignity, and care.

### Navigating the complexity of compassion

Healthcare providers and welfare professionals understand that compassion is vital for providing meaningful care, showing respect, and offering empathy to patients and their families, no matter their circumstances. According to Cullen et al. (2023), compassion is crucial for bridging gaps caused by societal stigma, especially in the treatment of individuals with mental health challenges. One nurse poignantly noted, “*Treat them like a human being…no matter what*,* they are somebody’s somebody. They are somebody*” [46].

In high-pressure settings like emergency departments, healthcare and welfare professionals stress the need for a compassionate and focused approach that humanises distressed service users [46]. Social workers and advocates emphasise recognising parents and young people in crisis as individuals beyond their challenges. A social worker in the study by Cullen et al. (2023) pointed:“*For me if I’m staying in a room with a patient*,* I try never to take a phone call if I’m sitting there having an assessment cause I want them to feel like they’re important*”. As Bai et al. (2020) proposed, normalising their circumstances helps service users feel recognised as individuals, promoting stronger connections and trust.

Atkin and Kroese (2022) emphasise the significance of approaching individuals, especially parents in distress, with empathy instead of viewing them merely as statistics. Advocates stress how small affirmations like “*You’re not a bad person*” can have a life-changing impact on parents dealing with overwhelming guilt and anxiety. When professionals treat individuals as mere names in documents instead of recognising them as whole human beings, they perpetuate feelings of worthlessness and disconnection [52]. For example, an advocate shared their experience of dehumanised care: “*I think it’s easy for people just to become a name on a piece of paper*,* and papers are passed on from one case to another*,* and one social worker to another*,* and they’re lost within that*”. Advocates frequently provide emotional support that other professionals can miss.

A study by Bai et al. (2020) suggested that treating service users with humanity involves normalising their experiences. Understanding that challenges are common to everyone, including professionals, can make individuals feel less alone in their struggles. A worker from Bai’s study emphasised that simply walking alongside someone, validating the difficulty of their situation, and reassuring them that these challenges are part of the human experience can foster a sense of shared humanity: “*I mean they’re still humans. They deal with stuff that us all humans deal with… they’re faced with a lot*,* and a lot of times it’s just a matter of having someone work with you alongside and just normalise that and making them feel like this is*,* this stuff is hard*” [44].

Compassionate care transcends mere practical assistance; it nurtures human connections that acknowledge the complete personhood of those in distress. This methodology strengthens relationships between professionals and service users, leading to better outcomes by establishing environments where individuals feel valued, respected, and understood.

Compassionate care creates a ripple effect through small acts of kindness and empathy, leading to profound transformations. Its influence extends beyond the individual receiving care. When healthcare providers, social workers, and advocates exhibit compassion, it not only improves the experience for service users but also positively impacts their families and the care providers themselves. Families feel supported, service users participate more meaningfully, and care providers experience both personal and professional growth as they enhance their emotional capacity. This interconnectedness emphasises the importance of embedding compassion in every aspect of care, ensuring that all parties are treated with humanity and respect.

All stakeholders recognise compassion as an acknowledgement of our shared humanity and respect for differences “*with a dedication to alleviating suffering*” [51]. Importantly, the key elements identified by all parties for delivering and receiving compassionate care are empathy, trust, and human connection (rapport).

### Crucial components of compassionate care

Taking a compassionate approach enables YP to establish stronger connections with healthcare providers, enhancing relationships that result in improved treatment and well-being outcomes. Nonetheless, YP recognises that empathy and trust play a vital role in this process connection.

YP identified important characteristics for fostering trust, including being heard, ensuring confidentiality, feeling secure, and experiencing authentic care [54]. They also emphasised that trust is gradually developed [54] as highlighted by one participant from their study: “*It took a while to build up the trust to be able to speak to her[…] and it only took a few weeks*,* because she came across as a very nice*,* genuine person to me*” (YP)”. The sense of psychological safety was also a contributing factor towards the development of the therapeutic alliance, as highlighted by another participant: “*I felt very much like all the things I was telling her were 100%confidential […] I felt very safe with her as my therapist*” [54].

Additionally, some YP have expressed feeling misunderstood, noting that their needs are frequently overlooked until they hit a crisis [65]. To overcome these negative experiences, YP emphasised the importance of empathy and professionalism, providing *‘parent-like’* support to facilitate trust and close professional connections and alliances [65]. A participant from the study by Loos et al. (2018) summarised: “*Good psychologists do more for you than they have to. They appreciate you*,* show empathy but they also disclose their mistakes to you; they are honest with you*,* normal psychologists listen to you (…) bad psychologists destroy you*”.

Parents expressed similar worries, emphasising the importance of compassionate care for their children’s health and emotional support. Many indicated that a deficiency in kindness or empathy makes them feel abandoned, isolated, undervalued, and unsupported until a crisis arises with their children [59]. This forces some parents to *‘fight the system’* and become proactive and assertive to get more appropriate care their children need [59]. A participant’s experience reflected this: “…i*f you are proactive and you push and fight and fight and fight*,* you will finally start to get some help*,* and that is the problem*” [59].

Healthcare professionals expressed feelings of anxiety, sadness, and uncertainty when engaging with adolescents dealing with complex challenges and trauma [51]. In therapy, practitioners present their true selves to connect with each patient’s individuality. Providers highlight the necessity of seeing clients as distinct individuals [51]. They acknowledged the uncertainty in their trauma work, which impacted their relationships. They emphasised the importance of recognising mistakes to foster trust, given that the therapeutic relationship was susceptible to disruptions due to the complexity of the young person’s trauma [51]. Additional benefits were also noticeable, including reducing the time required to establish therapeutic connections with their patients. For instance, one nurse explained a benefit: “*I think it’s accelerating the process of building rapport and trust*” [53].

Another study also noted that there are benefits of communication between healthcare professionals and patients as communication “*improves the transmission and retrieval of important clinical and psychosocial information*,* facilitates patient involvement in decision making*,* allows open discussion of benefits*,* risks*,* and barriers to adherence*,* [and] builds rapport and trust*” [48].

Social workers stress the significance of engaging with patients in community care to help them comprehend their situations and connect with suitable healthcare options. They consider this their top priority. Although they cannot address every issue in the emergency department, they strive to ensure patients receive the necessary support and assistance [46].

### Compassion-in-action

A study introduced compassion-in-action, a framework designed to assist individuals affected by trauma. It emphasises the significance of empathy and the practitioners’ “response-ability”—their obligation to actively engage with distressed clients [51]. This method highlights social justice and our collective humanity, fostering healing in diverse settings. It turns individuals into channels of care, demonstrating compassion to ease suffering.

Another study also explored compassion-in-action in online peer support settings [55]. Their findings suggest that compassionate action stems from exchanging personal experiences and vulnerabilities [55]. Peer supporters draw from their own painful experiences to offer compassion and encouragement to individuals facing suicidal thoughts or self-harm [55]. The lived experiences and supportive, nonjudgmental assistance they provide affirm help-seekers, enabling them to accept mental health challenges without stigma. These interactions cultivate emotional skills, assist in managing emotions, and strengthen community ties, underlining the importance of compassion, connection, and community for change and healing [55]. Peer supporters create a safe space through courtesy, rapport, listening, and empathy, reducing guilt and empowering individuals to address their mental health issues effectively.

### Complex interplay of facilitators and barriers to compassionate care

Various factors can facilitate or obstruct compassionate care on individual and organisational levels. The following section emphasises key factors influencing compassionate care, incorporating insights from healthcare professionals, advocates, and parents.

### Facilitators of compassionate care: individual versus organisational levels

Multiple elements are vital to improving the expression of compassionate care at the individual level. These include active listening, personal resilience and ethics.

Effective communication, particularly through active listening, is essential for providing compassionate care. Active listening consistently stood out among various participant groups as a key element. For example, parents appreciated healthcare professionals who demonstrated sensitivity and respect while discussing their child’s symptoms, as this helped alleviate feelings of blame and guilt. One parent noted, “*When providers respect us*,* ask for our opinions*,* and engage with our children in a friendly manner*,* we feel empowered*” [66].

The participants indicated that engaging in active listening is vital when addressing sensitive issues such as depression or suicidal thoughts. Respondents emphasised the need for healthcare professionals to listen without judgment to fully understand patients’ experiences and concerns. One nurse explained, “*Sometimes*,* it’s just effective listening and compassionate listening…because it’s not our job to judge people*,* it’s our job to care for them*” [66]. This highlights the essential role of communication in fostering compassionate care, especially in complex or emotionally charged situations.

Furthermore, the relationship among personal ethics, empathy, and resilience was recognised as crucial in promoting compassionate care. Advocates highlighted that resilience enabled them to manage their work’s moral and emotional difficulties while maintaining a compassionate approach [52]. One participant remarked that, despite the job’s difficulties, moral distress frequently resulted in personal growth and reinforced their dedication to compassionate care: “*Experiencing moral distress can facilitate personal and professional growth through the development of greater self-awareness*,* resilience*,* and moral resolve*” [57]. Peer and psychological support were seen as an effective strategy for alleviating burnout and enabling healthcare assistants to maintain their autonomy and compassionate outlook [56, 57].

Conversely, reducing supportive work environments, minimising power imbalances and opportunities for continuous professional development were identified as organisational facilitators of compassionate care.

According to the participants' feedback, a supportive workplace was identified as a key enabler. They emphasised that healthcare environments promoting teamwork, shared support, and joint accountability are beneficial for delivering compassionate care [57, 60]. Such supportive environments were deemed essential for helping healthcare professionals avoid burnout, enabling them to engage empathetically with patients, even under pressure. Participants highlighted that backing from colleagues and organisations empowered healthcare providers to uphold their compassionate demeanour, regardless of external challenges [56, 60, 63].

Furthermore, addressing power imbalances between healthcare, welfare providers, and patients emerged as a crucial facilitator. Participants noted that straightforward actions, such as providers introducing themselves by name, eased perceived hierarchies and fostered a more equitable therapeutic relationship [53]. A participant noted that recognising the provider’s name was crucial for fostering trust and enhancing the therapeutic process: “*Knowledge of a professional’s name enables the development of deeper therapeutic alliances while minimising differences between parties*” [53]. These findings indicate that minimising power dynamics can foster a more collaborative and empathetic healthcare setting.

Participants regarded continuous professional development and training as crucial for fostering compassionate care. Healthcare professionals, in particular, showed strong interest in training that would improve their capacity to support patients grappling with suicidal thoughts or self-harm. Significant training programmes aimed at enhancing empathy and interpersonal skills have positively influenced care. For example, a study revealed that a Compassionate Care Training initiative substantially raised compassionate care ratings among clinical supervisors [56]. The programme focused on empathy, interpersonal skills, and acceptance-based strategies, thus improving providers’ ability to connect with patients [56].

Moreover, it was recognised that training should extend beyond healthcare professionals. Some participants indicated that teaching parents about compassion, mindfulness, and acceptance could foster a more empathetic care environment for adolescents [55]. This highlights the need for a holistic approach to compassionate care, ensuring that both providers and caregivers have the crucial skills to offer emotional and psychological support.

## Barriers to compassionate care: personal versus systemic or structural factors

Compassionate care is essential for effective healthcare and welfare, yet numerous barriers hinder its consistent provision. These challenges include both personal and organisational factors, which will be examined in more detail in the following subthemes.

### Personal factors: poor communication and resistance to compassion

A study emphasised the desire of parents for improved communication with psychiatrists regarding their children, highlighting current gaps in understanding [62]. As one parent mentioned, “*Psychiatrists and nurses don’t make enough rounds and are careless. I think they can communicate more with patients*” [62]. Thus, communicating without empathy may fail to express compassionate care.

The study by Santos et al. (2023) highlights a major obstacle: healthcare professionals, caregivers, and young people frequently resist compassion. Some caregivers struggle to accept compassion from others. One participant explained, “*For me*,* it was difficult to let others be compassionate to me*” [60]. Self-compassion was also difficult, as highlighted by one caregiver: “*It is self-compassion that we have to work on. It is the more difficult one*” [60].

Caregivers observed that YP, particularly those with past trauma, frequently resisted compassionate methods. This resistance, rooted in their challenging experiences, made it difficult for caregivers to create the warmth and connection necessary for effective support. “*They are not used to this kind of intervention at all. In their family home*,* they went through violence and shouting*,* so it makes them a bit angry when we are compassionate towards them*” [60].

A potential way to address ‘the fear of compassion’ may involve compassionate accountability [45]. Survivors of trauma frequently feel that conventional accountability approaches disregard their viewpoints and may feel unsafe [45]. This may cause feelings of neglect, limited freedom, and increased insecurity, which could lead to re-traumatisation [45]. Compassionate accountability builds stronger connections between staff and youth, helping them manage trauma symptoms and encouraging young people to connect, collaborate, gain insights, and reflect on their treatment experiences and learning [45].

### Systemic and structural barriers

Alongside personal challenges, systemic barriers impeded the provision of compassionate care. Sowden et al. (2023) highlighted significant system-level issues, including access difficulties, misconceptions surrounding mental health conditions, and insufficient coordinated care. This disconnect between services led some caregivers to feel that their needs were insufficiently acknowledged or met supported. As noted in their study, “*lack of visibility of parents as carers and variability in joined-up care could act as potential barriers to optimal caregiver support*” [59].

Furthermore, organisational challenges, primarily stemming from insufficient staff and heavy workloads, impeded the provision of compassionate care. Study participants indicated that their workplaces experienced staffing shortages, which led to time constraints that hindered effective communication and timely patient care [58]. One psychiatrist explained, “*There are too many patients in the department and too few medical staff. When a patient appears suicidal or injures himself*,* we sometimes fail to detect it in time*” [62]. A nurse further emphasised, “*We do not have much time to communicate with patients and solve some of their psychological problems. Understaffing is a factor*,* and the second may be the division of labour is not optimised*” [62].

The research conducted by Santos et al. (2023) corroborated these results, especially in care homes. However, the structure of the Compassionate Mind Training (CMT) program posed challenges because of its considerable workload. As one caregiver remarked: “*These sessions*,* for some of us*,* were an extra working hour. For some people*,* it meant coming to work on days off or before working hours*,* when they would still have 8 hours of work after the session*” [60].

Additionally, many patients restricted the time medical staff had to provide personalised care. This issue was particularly emphasised by a mother in the Fu et al. (2021) study, who stated, “*I feel like they just come in the morning and ask if they have any questions. No one’s to chat with my child or to communicate with her*” [62]. A lack of time for significant discussions resulted in a misunderstanding of mental health conditions and treatments, particularly for families with children facing mild mental health difficulties who felt overlooked.

Moreover, the residential care staff faced noticeable work overload and compassion fatigue due to their demanding shifts. This hindered their full participation in reflective practices and training designed to enhance their compassion skills. “*The day is very busy*,* there were days that I could do the homework*,* others that I didn’t have the possibility to listen to the audios*” [60]. Subsequently, the emotional toll of continuously providing care in these environments led caregivers to exhibit distancing behaviours as they coped with exhaustion and emotional burnout.

## Discussion

This systematic review analysed 23 studies on compassionate care insights from YP, parents, caregivers, and healthcare professionals. The qualitative findings were categorised into two overarching themes: ‘Compassionate care is all about humanity’ and ‘the complex interplay of facilitators and barriers’. Only eight studies focused on young people’s experiences in community settings, primarily involving older adolescents, with younger adolescents or children largely absent. Furthermore, compassion was often not the main emphasis of these studies, reflecting findings from a scoping review that showed patients’ views on compassionate care are underrepresented [7]. While more research examined parents’ experiences, few addressed how they perceive compassion in real life. In contrast, studies involving healthcare and welfare staff provided deeper insights, unveiling various facilitators and barriers to implementing compassionate care.

### Compassionate care is all about humanity, the relationships and systems in which these exist

The review shows that YP and their parents prioritise compassionate care from healthcare providers. Participants stress that this type of care embodies humanity and fosters strong relationships. Existing research backs the notion that compassion from clinicians is essential for YP [67]. For instance, Stubbing and Gibson (2022) found that participants sought respect and validation from clinicians instead of feeling patronised. A robust therapeutic alliance is crucial for YP and parents in distress, as supportive relationships facilitate recovery [26, 68–70]. YP, parents, and caregivers favour professionals who listen attentively, recognise their individuality, and communicate openly [67]. When these criteria are fulfilled, service users notice a considerable enhancement in their overall care and views of their clinicians. These results align with the review’s findings, which stated that both verbal and non-verbal therapeutic skills are crucial for improving the overall care experience for service users [7].

This review’s findings also reveal that YP may be reluctant to accept compassion from health and welfare professionals because they fear it. These fears, in contrast to the protective effects of compassion, can increase susceptibility to psychological distress and may even worsen the effects of pre-existing mental health issues [71, 72]. A meta-analysis conducted by Kirby et al. (2019) revealed a moderate correlation between fears of self-compassion and receiving compassion and various mental health problems, including depression, anxiety, and stress, as well as general well-being. These fears are associated with vulnerability factors like self-criticism and shame, particularly in clinical populations with diagnosed mental health disorders [72].

Concerns about accepting compassion stem from past shame or trauma, causing people to remember fewer positive childhood experiences [73, 74]. Such experiences may create maladaptive social schemas, causing insecurity in social situations [75]. As a result, compassion can be seen as a threat. Individuals with Social Anxiety Disorder (SAD) may fear compassion due to feelings of unworthiness and the belief that needing compassion indicates weakness or leads to pity [13]. Consequently, those with SAD often engage in safety behaviours to conceal their discomfort and evade negative evaluation and support [76]. This suggests a link between fearing compassion and engaging in self-protective behaviours that prevent warmth from others. This observation may also apply to staff who care for young people, as referenced in this review. Nevertheless, the connection between the fear of compassion and the adoption of safety behaviours still requires in-depth investigation [77]. Cultural variations may also clarify anxieties related to fears of compassion.

A review highlighted that cultural differences influence how compassion is expressed and experienced [17]. In collectivistic cultures, such as those in East Asia, people frequently find it challenging to show compassion towards themselves and others [18]. This challenge arises from Eastern cultural norms that discourage seeking help, as it is viewed as a failure that can bring shame to oneself and those nearby [18]. Tight collectivist cultural norms can constrain the outward expression of compassion, yet they may promote greater self-compassion when compared to more relaxed cultural norms [19]. Cultural influences play a crucial role in shaping individual experiences of compassion. Consequently, when implementing Western compassion techniques in non-Western settings, it is vital to consider the pertinent cultural and social factors [18].

Furthermore, healthcare and welfare professionals agreed in this review that essential aspects of delivering compassionate care include treating service users with dignity, respect, and understanding while viewing them as human beings. This requires empathy and a genuine intent to alleviate their suffering. Similar conclusions emerged from another study involving healthcare professionals, which identified six fundamental components of compassionate care: emotional connection, a sense of worth, attention to the whole person, understanding, effective communication, and assistance with their practical needs [1].

Furthermore, the earlier scoping review and its revised edition concur that compassion arises within trusted relationships, characterised by one party’s suffering and the other’s wish to help ease that pain [7, 33]. These insights underscore the significance of compassionate care in promoting recovery and resilience through a therapeutic alliance. Compassionate care fosters positive therapeutic connections and is associated with improved satisfaction among service users, treatment adherence, and outcomes, particularly in mental health services [5, 70, 78].

On the other hand, a deficit of compassion may deter YP from help-seeking, which in turn can adversely impact their treatment results. Other systematic reviews have highlighted similar conclusions, showing that experiences of dehumanising care and a lack of respect and trust can cause YP to withdraw from treatment or support [79, 80]. However, self-compassion plays a crucial role for individuals in stressful situations, as it affects their mental health. As a result, one study suggested that the process of seeking help acts as a mediator between self-compassion and mental health outcomes [81].

In contrast, this review indicates that parents who exhibit lower levels of compassion are generally more proactive and determined regarding their children’s healthcare and welfare. Numerous studies underscore the significance of parental assertiveness in these contexts, suggesting that parents who encounter challenges in obtaining mental health services or support often adopt a more assertive approach [82]. This assertive behaviour appears to result in faster access to vital treatments, supporting previous research that underscores the essential role of parental advocacy in securing quality care for children [80, 83, 84].

However, it is crucial to recognise that many parents felt they had to take on this assertive approach, even if it was challenging for them, which supports earlier research findings [80, 83, 84]. Both YP and their parents emphasised the importance of feeling respected and valued in their engagements with healthcare and welfare professionals. This aligns with a broader perspective on compassion based on shared humanity, empathy, and dignity. Research shows that the essential elements of compassionate care lie in the mutual recognition of humanity between professionals and those they serve [5].

Earlier reviews indicated that compassionate care is predominantly conveyed in clinical environments through interpersonal relationships, especially in clinical communication [7, 33]. Clinicians’ readiness to engage with and be influenced by patients’ experiences is vital, necessitating vulnerability from both the clinicians and other professionals involved [27]. Conversely, this review emphasised the essential roles of social workers, advocates, and other welfare professionals in providing compassionate care, especially by maintaining dignity, which is characterised by care provided with warmth and empathy. This is in line with the recommendation of the Social Care Institute for Excellence (SCIE), which stated the following: “*People in these situations need care that is based on empathy*,* sensitivity*,* warmth*,* transparency and time. They don’t just need one talented and kind individual– they need an organisation that recognises and rewards compassionate care*.” [85].

Numerous sources concur that compassion encompasses empathy and a commitment to reducing suffering, but immediate support remains essential. In addition to empathy, the element of time is critical in providing compassionate care, as it fosters trust and relationships, allowing professionals to fully grasp the viewpoints of service users without hurrying through the care process [5]. This review reflects some YP’s opinions in this review that trust in professionals requires time, as a therapeutic alliance is not formed instantly. Consistently taking time to listen is essential for fostering authentic relationships and showing empathy [85]. Consequently, service users ought to receive support from committed and compassionate staff who operate within a culture that upholds integrity, respect, fairness, compassion, and trust as fundamental values [85]. Thus, understanding service users’ notions of compassion and integrating those insights into daily practice can be crucial for transforming healthcare and welfare, as compassion plays a vital role in ensuring quality care and strong health and support systems [70].

The findings of this review, combined with the definitions of compassionate care, suggest that time should be a part of its definition. Hence, we propose the following definition: “***Compassionate care requires a recognition and understanding of the suffering of others***,*** coupled with a genuine intention to alleviate that suffering in a timely and responsive manner***”.

The above-mentioned definition encompasses empathy, where one experiences another’s suffering and extends to motivate immediate actions that offer support and relief during critical times. Compassion demands sensitivity to others’ emotions while dedicating oneself to providing understanding, kindness, and help at the right moments to significantly enhance their well-being.

This method requires healthcare providers to actively engage with patients as unique individuals, recognise their specific physical, emotional, and psychological needs, and respond promptly with warmth and empathy to deliver patient-focused care that reduces distress and builds trust. This strategy not only boosts patient satisfaction and speeds up recovery but also fortifies the therapeutic relationship by honouring each patient’s dignity, providing timely interventions, and appreciating the human aspect of healthcare.

### The interplay between facilitators and barriers to compassionate care

This systematic review revealed several factors that either enhance or obstruct compassionate care in community healthcare and welfare settings. Key facilitators include nurturing empathy, establishing supportive work environments, encouraging effective communication, and promoting active listening. Furthermore, tackling power dynamics and providing appropriate training enhanced compassionate care. These insights align with previous research highlighting the critical role of communication and understanding in building compassionate relationships between healthcare providers and patients [27].

On the other hand, this review found that obstacles such as communication barriers, language issues, inadequate staffing, time pressures, and heavy workloads hinder compassionate care. Compassion fatigue and difficulties in forming emotional connections exacerbate these issues, especially in high-stress settings. Professionals in emotionally charged environments face heightened risks of vicarious trauma and burnout [35, 70]. Previous research indicates that burnout and moral injury significantly threaten mental health professionals, leading to emotional detachment and reduced compassion [70].

The review highlights that a lack of organisational support and training affects both staff well-being and the quality of care. Healthcare professionals who work directly with service users often miss out on vital training related to conflict resolution and managing serious mental health challenges [70]. Consequently, this lack of training can lead to increased burnout and stress due to unfavourable working conditions, creating negative attitudes towards service users [1].

The review suggests that focusing exclusively on diagnostic factors in mental health can lead to negative perceptions of service users, which in turn can cause dehumanisation and less compassionate care, as noted in another review [7]. Additionally, an emphasis on meeting targets and addressing legal concerns can hinder staff’s ability to offer care [70]. When faced with repeated refusals of essential treatment, staff might develop emotional detachment or adopt a mindset of “normalisation of deviance,” where harmful practices become accepted due to systemic constraints [70].

The findings highlight the importance of balancing workload with autonomy and control, nurturing supportive workplace relationships, and enhancing employee well-being. Creating opportunities for reflection and self-care, and linking theoretical knowledge to hands-on experiences are essential components of providing compassionate care [1]. Yet, ongoing challenges like staff shortages, time limitations, and weak team cohesion pose considerable obstacles, especially in environments marked by adverse workplace cultures and resistance to change [33].

While organisational factors primarily restrict compassionate care, individual and team-level elements are also influential. Personal stressors and weak team bonds can hinder compassionate interactions [1]. Therefore, accounting for these multi-level factors is essential when creating interventions to enhance compassionate care in healthcare environments. Addressing these obstacles requires a holistic strategy that integrates individual, team, and systemic perspectives. Although failures to act with compassion occur at the interpersonal level, systemic and structural factors undermine mental health staff’s ability to provide compassionate care [70].

In one study, staff members identified essential elements that promote compassionate care, including individual factors such as effective coping strategies for workplace stress, team dynamics like feeling connected with colleagues, and organisational elements like feeling valued in their roles [1]. Conversely, barriers to delivering compassionate care, primarily concerning organisational aspects like insufficient resources and lack of practical support, were identified by staff [1]. Some individual challenges, like personal life difficulties and team-related issues, like insufficient connection with team members, were also noted [1].

Nonetheless, several studies have highlighted multiple barriers to delivering compassionate care, including a lack of resources such as time constraints, heavy workloads, staff shortages, and strained relationships among colleagues [1, 27, 33]. The findings of this review align closely with existing literature. Given that established research shows compassion fatigue affects both individual and organisational factors, it is probable that personal, group, and organisational elements play vital roles in facilitating or hindering the provision of compassionate care [1, 27].

### Strengths and limitations

This review effectively synthesises the experiences of CYPs and their families regarding compassionate care, representing the first comprehensive account of these experiences in healthcare and welfare. However, many studies focusing on young people, parents, or caregivers did not highlight their experiences with compassionate care. Additionally, the review features a robust and well-organised search strategy that yielded consistent articles from further literature searches. We maintained methodological rigour by adhering to PRISMA standards and the original PROSPERO protocol.

There are limitations to this review. Firstly, as all except three analysed studies were from English-speaking countries, this may have limited the range of cultural contexts and healthcare systems included in this review. Mental health and welfare frameworks vary widely between countries, impacting the understanding of how compassionate care is understood and delivered. These cultural variations not only influence care delivery but also affect perceptions of compassion, which may result in varying study outcomes. Moreover, disparities in healthcare infrastructure influence accessibility and the quality of services offered. These differences may lead to inconsistent results in studies, as availability of mental health services and interpretations of compassionate care vary according to systemic frameworks.

Secondly, qualitative research offers valuable insights into individual experiences but tends to be context-dependent, challenging cross-setting comparisons. The diverse qualitative methodologies encompassed in this review further complicate comparisons between studies and may introduce interpretive differences in how compassionate care is conceptualised and analysed. For example, thematic analysis provides general insights into compassionate care patterns, but it may not fully capture the richness of personal experiences as effectively as interpretative phenomenological analysis (IPA), which emphasises lived experiences from an individual viewpoint. While case study methods offer detailed examinations of particular contexts, they can restrict the broader applicability of findings. Studies using mixed methods that integrate various qualitative approaches deepen understanding but might face challenges in integrating different views on compassion. Additionally, review articles and discussion studies offer meaningful theoretical insights but are fundamentally influenced by the selection and interpretation of existing literature, rather than direct experiences from participants. The qualitative studies featured in this review also had small sample sizes, which may complicate the generalisation of their findings to the broader population. On the other hand, variations in study designs included in this review can also affect the measurement and interpretation of compassionate care. Moreover, the time frame of the studies adds variability, as changing policies and societal dynamics affect perceptions and implementations of compassionate care.

Lastly, despite efforts to uncover grey literature, time constraints for this project resulted in limited inclusion of grey literature largely sourced from the ASSIA database.

### Recommendations for future research

This review highlights a significant lack of experiential data on compassionate care from CYP and their parents or carers in the existing literature. This gap is especially apparent regarding males, fathers, and younger children and adolescents, signalling a need for further research. Therefore, more qualitative studies are needed to explore the perspectives of these stakeholders, thereby deepening our understanding of compassionate care in diverse community settings. Future research should emphasise cross-cultural comparative studies to enhance our understanding of compassionate care and create culturally adaptive models that cater to diverse healthcare environments.

## Conclusion

This review identified 23 studies that underwent thematic narrative synthesis, revealing two overarching themes: ‘Compassionate care is all about humanity’, which emphasises human connections and compassion, and ‘the complex interplay of facilitators and barriers’ that can enable and/or hinder compassionate care. This review highlights the intricate nature of compassionate care in healthcare and welfare settings, emphasising the role of human relationships and compassion in improving outcomes for YP and their families. The findings stress the need to include time in the definition of compassionate care, as time, besides empathy and willingness to alleviate suffering, is a crucial component of compassionate care. Moreover, the review also stressed a need for a firm commitment to compassionate care, particularly in mental health services, where its absence can significantly impact help-seeking behaviours and recovery trajectories.

Healthcare professionals aim to deliver compassionate care; however, systemic issues like insufficient staffing, time limitations, and emotional exhaustion can obstruct these efforts. Moreover, a lack of compassion in healthcare interactions may cause parents and YP to feel isolated and under-supported, negatively affecting their treatment outcomes and future help-seeking behaviours.

To overcome these obstacles, the review outlines several enablers of compassionate care: training, nurturing work environments, effective communication, and initiatives to reduce power dynamics. Transforming these aspects through systemic changes—like policy reforms, enhanced support to combat burnout, and improved communication methods—can establish a more compassionate healthcare culture.

This review’s findings highlight that nurturing a compassionate culture in healthcare necessitates a well-rounded strategy that includes both personal commitment and institutional backing. By emphasising empathy, resilience, connection, and timely support, healthcare and welfare systems can convert compassionate care from an abstract concept into a regular practice, thereby enhancing care experiences and outcomes for everyone involved.

## Appendix 1: CASP quality assessment of included articles


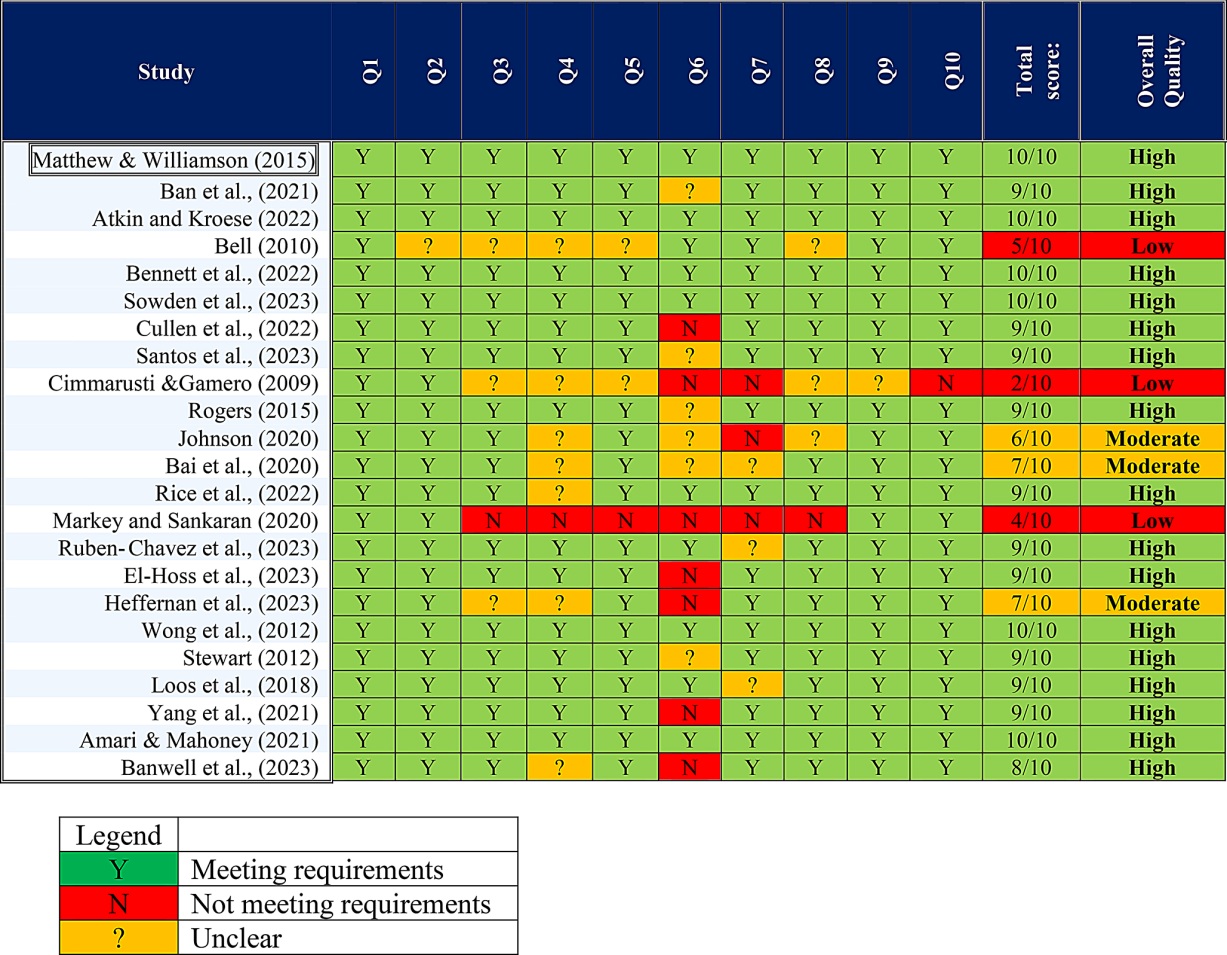


## Data Availability

No datasets were generated or analysed during the current study.
